# Recent Advances in Functional Nanomaterials for Electrochemical Sensors and Biosensors

**DOI:** 10.3390/molecules28196798

**Published:** 2023-09-25

**Authors:** Liqiang Luo

**Affiliations:** Department of Chemistry, College of Sciences, Shanghai University, Shanghai 200444, China; luck@shu.edu.cn

## 1. Introduction

Considering the unique advantages of the quantum size, volume, surface, and macroscopical quantum tunnel effects, nanomaterials have been paid increasing attention for various applications in environmental, medical, biological, and chemical analyses in recent decades. Recently, nanomaterial-based electrochemical sensors and biosensors have been widely focused on, due to their outstanding properties in terms of conductivity, selectivity, and biocompatibility, as well as their cost-effectiveness [1,2]. In this regard, different functional nanomaterials (such as carbon nanotubes, metal nanoparticles, metal oxides, metal nanoclusters, and metal–organic frameworks) have been synthesized and applied in electrochemical sensors and biosensors.

In this Special Issue, titled “Recent Advances in Functional Nanomaterials for Electrochemical Sensors and Biosensors”, eight contributions, including six original research papers and two review papers, were collected to showcase the recent advances in various functional nanomaterials for electrochemical sensors and biosensors.

## 2. Results

In one review, Soto and Orozco give a comprehensive review on recent advances in the applications of hybrid nanobioengineered nanomaterials in ultrasensitive electrochemical biosensors, mainly based on novel organic–inorganic hybrids with enhanced electrophysicochemical properties, including some recently engineered organic–inorganic metallic, silicon, carbonaceous, and polymeric nanomaterials [3]. The limitations and opportunities of hybrid nanobioengineered nanomaterials have been discussed from the point of view of current nanotechnology. Future considerations for advancing their use in enhanced electrochemical platforms have also been indicated.

In another review, Amen et al. present an overview of metal-oxide semiconductor field effect transistor biosensors for point-of-care testing devices [4]. Various metal oxide semiconductor materials (gallium oxide, copper oxide, cerium oxide, vanadium oxide, titanium dioxide, indium oxide, indium tin oxide, zinc oxide, and tin oxide) have been applied in field effect transistor-based biosensors, due to their superior electrical characteristics, chemical stability, and ease of fabrication. The analytical validation and regulatory approval of point-of-care testing devices have been evaluated. Finally, the adoption barriers, limitations, challenges, and future perspectives were discussed and addressed.

Wang et al. developed a simple strategy to prepare a biofilm reactor sensor for the determination of biochemical oxygen demand [5]. In their work, the microorganisms in fresh water were domesticated by artificial seawater with different salinity gradients successively to prepare the biofilm reactor sensor, which exhibited an efficient ability to degrade a variety of organic substances. The linear range of biochemical oxygen demand determination by the biofilm reactor sensor was 1.0–10.0 mg/L, with a detection limit of 0.30 mg/L. Therefore, their creative work provide a universal method for the determination of biochemical oxygen demand of both fresh water and seawater, overcoming the limitation of traditional biochemical oxygen demand sensor, which can only be used in one kind of water body due to poor environmental adaptability.

Li et al. constructed an ultrasensitive electrochemical biosensor for glypican-3 detection using the hemin-reduced graphene oxide-palladium nanoparticles and nanozyme-enhanced silver nanoparticles deposition signal amplification strategy [6]. In their work, the amount of deposited Ag nanoparticles, which was derived from the amount of glypican-3, was quantified by differential pulse voltammetry. Under optimal conditions, the current response was logarithmically linear, with a glypican-3 concentration in the range of 0.01–10.0 µg/mL, and the limit of detection was 3.30 ng/mL. They offered an effective strategy for the determination of glypican-3 with potential clinical applications.

Wang et al. synthesized bismuth nanoparticles decorated with one-dimensional honeycomb-like carbon nanofibers for the sensitive determination of nitrite [7]. In their work, one-dimensional honeycomb-like carbon nanofibers were synthesized with the electrospinning technique, on which highly dispersed bismuth nanoparticles were grown. The obtained bismuth/honeycomb-like carbon nanofibers exhibited excellent electrocatalytic activity towards nitrite oxidation. The fabricated sensor showed a wide range of 0.1–800 µM, with low detection limit of 19 nM. The excellent properties of the sensor were attributed to the unique structure of honeycomb-like carbon nanofibers, which can not only enhance the active area of material, but also regulate the growth of bismuth nanoparticles on honeycomb-like carbon nanofibers. The sensor has been successfully used for nitrite detection in food and environment samples, indicating its feasibility for further practical applications.

Zhang et al. synthesized porphyrin-functionalized carbon quantum dots through electrostatic interaction by a simple mixing method, which was used as a novel luminescent nanocomposite for the sensitive detection of copper ions in the Cu^2+^ quenching luminescence of functionalized carbon quantum dots [8]. The constructed sensor could sensitively determine copper ions in the range of 10 nM to 10 µM, and the detection limit was 2.78 nM. The proposed biosensor shows a strong ability to analyze real samples, and has a significant signal response in the serum samples, which is expected for early diagnosis of diseases and environmental monitoring.

Wang et al. developed a novel electrochemical sensor by electrodepositing polyglycine/reduced graphene oxide on a glassy carbon electrode for the simultaneously selective and sensitive determination of xanthine and hypoxanthine [9]. By taking advantage of the distinguished electrocatalytic performance of polyglycine and the good electrical conductivity of reduced graphene oxide, under optimal conditions, a linear response was acquired for the simultaneous determination of xanthine and hypoxanthine in the contration range of 1–100 µM. The proposed sensor exhibited high sensitivity, satisfying anti-interference ability, good stability, and reproducibility.

Huang et al. established a rapid detection method for rutin in food using nitrogen-doped carbon quantum dots as the fluorescent probe [10]. In their work, nitrogen-doped carbon quantum dots were prepared via a single-step hydrothermal process, using citric acid as the carbon source and thiourea as the nitrogen source. The synthesized nitrogen-doped carbon quantum dots showed amorphous carbon structures with good water solubility and optical property, with a quantum yield of 24.1%. In 0.1 M phosphate-buffered solution (pH 7.0), rutin had a strong fluorescence-quenching effect on nitrogen-doped carbon quantum dots. The method showed good linearity when the concentration of rutin was in the range of 0.1–400 µg/mL, and the detection limit was 0.033 µg/mL. The proposed method is simple, rapid, and sensitive, and can be used for the rapid detection of rutin in food.

## 3. Conclusions

In conclusion, this Special Issue aims to provide an overview of recent progress in applications of functional nanomaterials in electrochemical sensors and biosensors. As discussed above, various functional nanomaterials (such as organic–inorganic metallic, silicon, carbonaceous, and polymeric nanomaterials) have been synthesized and applied in constructing different electrochemical sensors and biosensors in the fields of environmental, biochemical, and pharmaceutical analyses. In fact, many techniques have been developed to synthesize functional nanomaterials to improve the sensitivity, biocompatibility, stability, and repeatability of electrochemical sensors and biosensors, including solvent-thermal, hydro-thermal, chemical vapor deposition, electrodeposition, and electrospinning methods. Despite some barriers and challenges, we believe that more functional nanomaterials with controllable and adjustable morphologies, structures, and properties will be designed, synthesized, and applied in fabricating electrochemical sensors and biosensors.

## References

[1] Wongkaew N., Simsek M., Griesche C., Baeumner A.J. (2019). Functional Nanomaterials and Nanostructures Enhancing Electrochemical Biosensors and Lab-on-a-Chip Performances: Recent Progress, Applications, and Future Perspective. Chem. Rev..

[2] Hsiao W.W.W., Fadhilah G., Lee C.C., Endo R., Lin Y.J., Angela S., Ku C.C., Chang H.C., Chiang W.H. (2023). Nanomaterial-Based Biosensors for Avian Influenza Virus: A New Way Forward. Talanta.

[3] Soto D., Orozco J. (2022). Hybrid Nanobioengineered Nanomaterial-Based Electrochemical Biosensors. Molecules.

[4] Amen M., Pham T., Cheah E., Tran D., Thierry B. (2022). Metal-Oxide FET Biosensor for Point-of-Care Testing: Overview and Perspective. Molecules.

[5] Wang L., Lv H., Yang Q., Chen Y., Wei J., Chen Y., Peng C., Liu C., Xu X., Jia J. (2022). A Universal Biofilm Reactor Sensor for the Determination of Biochemical Oxygen Demand of Different Water Areas. Molecules.

[6] Li G., Wang B., Li L., Li X., Yan R., Liang J., Zhou X., Li L., Zhou Z. (2023). H-rGO-Pd NPs Nanozyme Enhanced Silver Deposition Strategy for Electrochemical Detection of Glypican-3. Molecules.

[7] Wang F., Li Y., Yan C., Ma Q., Yang X., Peng H., Wang H., Du J., Zheng B., Guo Y. (2023). Bismuth-Decorated Honeycomb-like Carbon Nanofibers: An Active Electrocatalyst for the Construction of a Sensitive Nitrite Sensor. Molecules.

[8] Zhang X., Hou X., Lu D., Chen Y., Feng L. (2023). Porphyrin Functionalized Carbon Quantum Dots for Enhanced Electrochemiluminescence and Sensitive Detection of Cu^2+^. Molecules.

[9] Wang T., Zhang L., Zhang C., Deng D., Wang D., Luo L. (2023). Simultaneous Determination of Xanthine and Hypoxanthine Using Polyglycine/rGO-Modified Glassy Carbon Electrode. Molecules.

[10] Huang Y., Si X., Han M., Bai C. (2022). Rapid and Sensitive Detection of Rutin in Food Based on Nitrogen-Doped Carbon Quantum Dots as Fluorescent Probe. Molecules.

